# Choroidal hypertransmission width on optical coherence tomography: a prognostic biomarker in idiopathic macular hole surgery

**DOI:** 10.1007/s00417-024-06427-8

**Published:** 2024-03-26

**Authors:** Micol Alkabes, Alessandro Rabiolo, Andrea Govetto, Paolo Fogagnolo, Stefano Ranno, Mattia Marchetti, Filippo Frerio, Davide Wild, Valentina Gatti, Andrea Muraca, Stefano De Cillà

**Affiliations:** 1 Department of Ophthalmology, University Hospital Maggiore della Carità, Corso Mazzini 18, 28100 Novara, Italy; 2 Department of Health Sciences, Università del Piemonte Orientale “Amedeo Avogadro”, Novara, Italy; 3 Ophthalmology Department, Circolo and Fondazione Macchi Hospital, ASST Sette Laghi, Varese, Italy; 4 Eye Clinic, San Paolo Hospital, University of Milan, Milan, Italy

**Keywords:** Macular hole, Pars plana vitrectomy, Minimum diameter, Prognostic factor, Visual acuity, Surgical outcomes

## Abstract

**Purpose:**

To test the hypothesis that optical coherence tomography (OCT) choroidal hypertransmission width (CHW) is a prognostic biomarker in idiopathic macular hole (MH) surgery

**Methods:**

Retrospective cohort study of consecutive patients undergoing successful pars plana vitrectomy for idiopathic MH. We collected demographic, clinical, and OCT variables at the preoperative and last available visits. Two investigators assessed the following OCT parameters: MH minimum diameter, base diameter, CHW, ellipsoid zone, and external limiting membrane status (absent vs. present). Delta CHW was calculated as the difference between CHW and MH minimum diameter. Linear models were used to investigate factors associated with postoperative best-corrected visual acuity (BCVA) and BCVA change.

**Results:**

Thirty-six eyes (36 patients) with a median (interquartile range (IQR)) follow-up of 9 (8–11) months were included. The median BCVA (IQR) improved from 0.75 (1–0.6) logMAR preoperatively to 0.2 (0.6–0.1) logMAR at the last visit (*p* < 0.001). Preoperative MH minimum diameter (for a 10-μm increase, estimate (standard error (SE)): 0.009 (0.003) logMAR, *p* = 0.003), base diameter (for a 10-μm increase, 0.003 (0.001) logMAR, *p* = 0.032), CHW (for a 10-μm increase, 0.008 (0.002) logMAR, *p* < 0.001), and delta CHW (for a 10-μm increase, 0.013 (0.005) logMAR, *p* = 0.009) were significantly associated with postoperative BCVA. The proportion of variance explained was the highest for MH CHW (*R*^2^ 0.35), followed by minimum MH diameter (*R*^2^ 0.24), delta CHW (*R*^2^ 0.19), and MH base diameter (*R*^2^ 0.14). None of the study variables was associated with delta BCVA.

**Conclusion:**

Preoperative CHW is associated with postoperative visual acuity in patients undergoing successful idiopathic MH surgery and may be a useful OCT prognostic biomarker.

**Supplementary Information:**

The online version contains supplementary material available at 10.1007/s00417-024-06427-8.


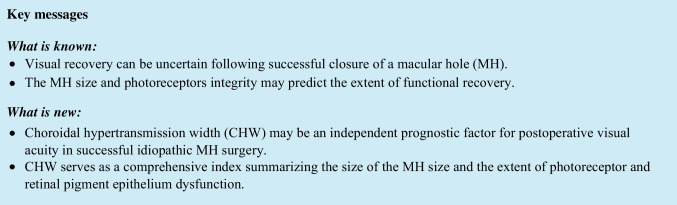


## Introduction

Idiopathic full-thickness macular hole (MH) is an acquired macular disease characterized by a defect in the neurosensory retina, resulting from tractional forces exerted by the inner limiting membrane (ILM) and vitreous. Clinical examination, including dilated fundus examination, is of paramount importance in diagnosing MH. In the past two decades, optical coherence tomography (OCT) has emerged as a valuable tool in diagnosing, staging, and monitoring idiopathic MH.

Pars plana vitrectomy has become the treatment of choice for MH. Although modern surgical techniques have led to remarkably high anatomic success rates, functional recovery may be uncertain, even after successful MH closure. There has been, therefore, interest in identifying factors that predict the extent of visual recovery in patients undergoing MH surgery. The size of the MH is a well-established predictive factor, and previous studies have shown that larger MHs have lower anatomical success rates and worse visual prognosis [1–5]. There are various methods to estimate MH size, but the minimum horizontal diameter is the most commonly used parameter because it is simple to measure, holds greater prognostic relevance than other metrics (i.e., base diameter), and is at the foundation of the current staging system [6, 7]. Still, there is considerable variability in visual recovery even among MHs of similar size due to the variable extent of photoreceptor damage [8].

The postoperative restoration of the ellipsoid zone (EZ) and external limiting membrane (ELM) are established markers to determine the degree of visual recovery [9–11]. Several studies have evaluated associations between preoperative EZ-ELM status and postoperative visual acuity with controversial results [12–15]. Assessing the integrity of outer retinal hyperreflective bands preoperatively may be arduous due to the distorted macular anatomy at the hole margins [8]. Furthermore, EZ-ELM defects tend to become progressively smaller or even be completely restored after successful MH closure, suggesting that abnormalities in outer retinal bands may, to some extent, reflect retinal disorganization rather than irreversible photoreceptor damage [9, 15]. Consequently, other biomarkers acting as surrogate measures of photoreceptor integrity have been investigated to better predict postoperative visual outcomes [8].

Recently, choroidal hypertransmission has gained popularity as a prognostic biomarker in age-related macular degeneration (AMD) [16, 17]. Choroidal hypertransmission occurs due to increased light penetrance into the choroid. In AMD, this is believed to be caused by the loss of highly scattering retinal pigment epithelium (RPE) melanin granules and, in more advanced stages, the loss of RPE itself [17]. Choroidal hypertransmission may also be found in other conditions. Vance and colleagues [18] observed transient hypertransmission defects in the acute stages of multifocal choroiditis (MFC), suggesting that these may occur due to photoreceptors and RPE impairment by subretinal inflammatory material. Palmieri et al. [19] found choroidal hypertransmission in highly myopic eyes with outer lamellar MH, with hypertransmission areas matching areas of EZ loss. Mehta et al. [20] investigated choroidal hypertransmission in patients undergoing MH surgery and found that choroidal hypertransmission width (CHW) was correlated with postoperative visual acuity.

In our observations, choroidal hypertransmission in patients with MH often extends far beyond the margins of the hole, and we believe that the choroidal hypertransmission width (CHW) may be a surrogate measure for the status of photoreceptors. This study aims to test the hypothesis that CHW, as measured with OCT, is a biomarker associated with visual outcomes after idiopathic MH surgery.

## Materials and methods

In this single-center retrospective cohort study, we evaluated consecutive patients who underwent a 25G standard three-port pars plana vitrectomy (PPV) for idiopathic MH between 2019 and 2021 at the Azienda Ospedaliero-Universitaria Maggiore della Carità, Novara, Italy. This study adhered to the tenets of the Declaration of Helsinki and received approval from our hospital institutional ethics committee. Written informed consent was obtained from all study participants.

Inclusion criteria were the presence of an idiopathic MH of any size successfully treated with pars plana vitrectomy and a minimum follow-up of 6 months. Successful treatment was defined as postoperative MH closure as evaluated with dilated fundus examination and OCT. The exclusion criteria were (1) impending MH (stage 1); (2) traumatic MH; (3) MH persisting after surgery; (4) an axial length greater than 26.5 mm; (5) the presence of a posterior staphyloma; (6) patients who underwent ILM peeling using the inverted flap technique; and (7) patients with a history of past ocular surgery, except for uncomplicated phacoemulsification. Patients with diabetes mellitus or a history of other ocular and systemic conditions potentially influencing anatomical and visual functions were also excluded. In cases where both eyes of a patient were eligible, only the first operated eye was included in the study.

Being a retrospective study, the investigations and follow-up times were predetermined but were conducted at the discretion of the treating surgeons. Overall, the following examinations were performed at the baseline preoperative visit: best-corrected visual acuity (BCVA) on Snellen charts, anterior segment slit-lamp examination, dilated fundus examination, SD-OCT of the macula (Spectralis HRA-OCT; Heidelberg Engineering GmbH, Heidelberg, Germany), and optical biometry (Zeiss IOL Master, Zeiss, Jena, Germany). At postoperative visits, patients underwent BCVA, anterior and posterior segment slit-lamp examinations, and SD-OCT imaging.

### Surgical technique

Two experienced vitreoretinal surgeons (SDC and MA) performed all surgeries with a standardized technique. Three-port 25G PPV was performed with the Stellaris Elite vitrectomy system (Bausch & Lomb, Laval, Canada). After inducing a posterior vitreous detachment (PVD) as needed, the surgeon performed epiretinal membrane (ERM) and inner limiting membrane (ILM) peeling using dedicated forceps and 0.2 mL of blue dye (Membrane-Blue; DORC, Zuidland, The Netherlands). ILM peeling was extended over an area of 2- to 3-disc diameters. Following the ERM/ILM peeling, a fluid/air exchange was performed with the aspiration of residual fluid through the MH and then injected with 20% sulfur hexafluoride (SF6) as a tamponading agent. Patients were instructed to maintain a prone position for two days postoperatively. In the case of preoperative cataracts, phacoemulsification with intraocular lens implants was performed simultaneously, based on surgeons’ and patients’ preferences.

### Optical coherence tomography (OCT) parameters

**Fig. 1:**
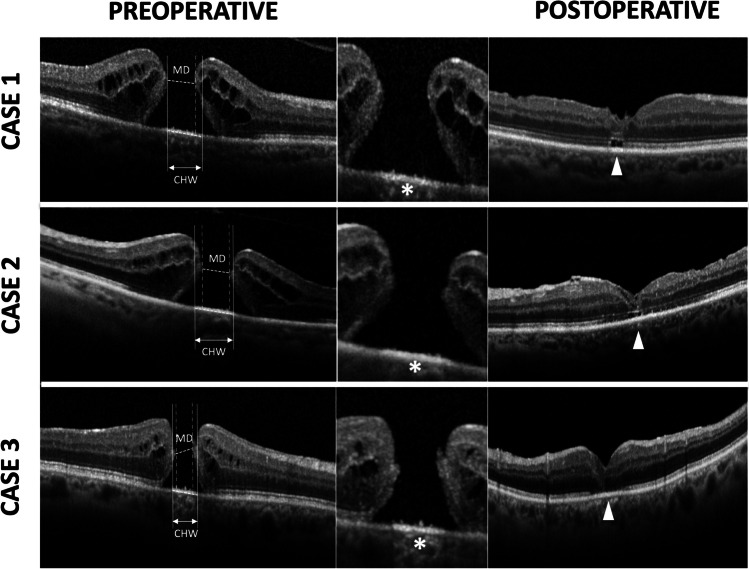
Spectral domain optical coherence tomography (SD-OCT) in three patients undergoing macular hole (MH) surgery. The *left column* shows preoperative measurements made on SD-OCT scans: MH minimum diameter (MD) was determined as the minimum distance between hole margins; choroidal hypertransmission width (CHW) was measured at the hyperreflective line corresponding to the retinal pigment epithelium (RPE). Delta CHW was calculated as the difference between CHW and MD. The *central column* shows magnified details of both preoperative EZ-ELM status at the MH margins and CHW (white asterisk). The *right column* shows postoperative macular hole closure with interrupted EZ-ELM (white arrowhead). ELM, external limiting membrane; EZ, ellipsoid zone; RPE, retinal pigment epithelium

The quality of OCT images was reviewed, and poor-quality images were excluded. For each patient, a single horizontal 9-mm high-quality line scan (consisting of 100 averaged frames) that displayed the greatest MH dimension was acquired to determine OCT parameters. Two investigators (MM and FF) independently measured the following parameters at the preoperative scans: MH minimum diameter, base diameter, and CHW (Fig. 1).

To enhance accuracy, acquired OCT scans were then analyzed with a zoom factor of 800% included in the corresponding software with a resolution of 3.87 μm/pixel axially and 11.1 μm/pixel laterally. Quantitative measurements were obtained using the built-in caliper of the Spectralis OCT device software. The qualitative analysis included the EZ and ELM status, categorizing them as present or absent. MH stage was classified according to the International Vitreomacular Study group subclassification for MH [6].

### Statistical analysis

The distributions and normality of all study variables were assessed using frequency histograms and quantile-quantile plots. Mean (±standard deviation (SD)) and median (interquartile range (IQR)) were reported for normally and non-normally distributed continuous variables; frequency and proportions were reported for non-continuous variables. Prior to statistical analysis, Snellen visual acuity values were converted to the logarithm of the minimum angle of resolution (logMAR) scale.

Relationships among minimum diameter, base diameter, and hypertransmission diameter were investigated with bivariate plots. Delta CHW was defined as the difference between MH minimum diameter and CHW, representing the retina segment with CHW extending over the MH aperture.

Linear regression models were used to evaluate demographic, clinical, and OCT preoperative factors associated with (i) postoperative BCVA at the last available follow-up visit and (ii) BCVA change, defined as the difference between the last available follow-up visit and the preoperative visit. The study covariates included preoperative age, gender, preoperative lens status (i.e., phakic vs. pseudophakic), preoperative BCVA, MH stage, preoperative ELM and EZ status (present vs. absent), preoperative cysts at the MH margins, preoperative MH minimum diameter, base diameter, CHW, delta CHW, and follow-up time. Relationships and degree of correlations among study covariates were visually inspected with hierarchical cluster analysis based on Spearman |rho| coefficient values (Supplementary Figure 1). Linear regression analysis was performed between each study covariate and dependent variable. Point estimates, standard errors (SEs), *p*-values, and *R*^2^ coefficients were reported.

Given the high correlation among MH minimum diameter, base diameter, and CHW, simultaneous incorporation of these variables in multivariable analyses was not feasible, to avoid collinearity. Consequently, we performed separate univariable models for each covariate. Collinearity is a known source of unstable regression estimates and large standard errors. We also ran linear regression models after standardizing all dependent and independent variables to evaluate which OCT parameters had the greatest impact on explaining postoperative BCVA and BCVA change. Standardization facilitates the comparison of the magnitude effect of different model covariates by putting them on the same scale (i.e., zero mean and unit SD). Also, we ran multivariable models containing both preoperative MH minimum diameter and delta CHW to test the hypothesis that delta CHW is associated with visual acuity recovery while accounting for MH size. As we found that delta CHW was mildly correlated with MH minimum diameter, we also ran multivariable models after normalizing delta CHW values to make them completely independent from the MH size. Normalization was performed by linearly regressing delta CHW against MH minimum diameter. The observed delta CHW values were then divided by their corresponding predicted values. Normalized delta CHW was uncorrelated to the minimum MH diameter (Supplementary Figure 2). Ultimately, a standardized multivariable model was run, which included other variables previously associated with postoperative visual acuity (i.e., preoperative BCVA, preoperative absence of EZ, and follow-up duration). In this model, all variables were standardized except for the categorical variable indicating the absence of EZ.

To adjust for the uneven postoperative follow-up, follow-up time was included as a model covariate along with the covariates of interest in all models. All the statistical analyses were conducted using the open-source software R (R Foundation for Statistical Computing, Vienna, Austria). All tests were 2-tailed, and *p*-values ≤0.05 were considered statistically significant.

## Results

**Fig. 2:**
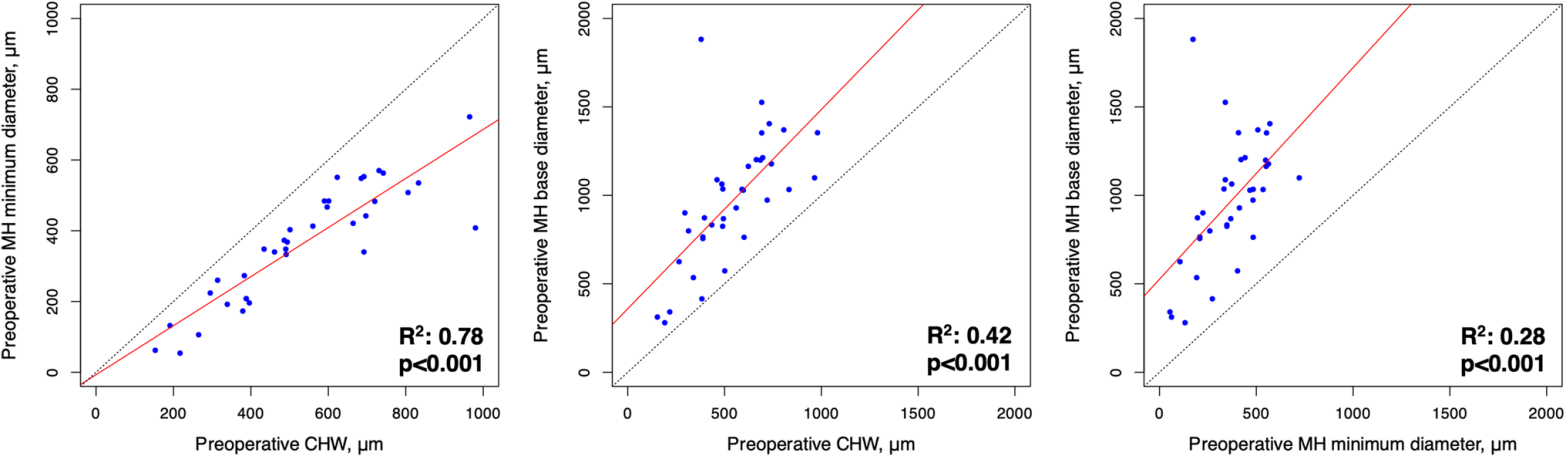
Bivariate plots of preoperative MH minimum diameter and CHW (*left panel*), CHW and base diameter (*middle panel*), and MH minimum diameter and base diameter (*right panel*). Solid red and dotted black lines indicate regression and equivalence lines, respectively. CHW, choroidal hypertransmission width; MH, macular hole

Thirty-six eyes from 36 patients, with a median (interquartile range (IQR)) follow-up of 9 (8–11) months, were included in the study. Table 1 summarizes the demographic and ophthalmic data of the study participants. The median BCVA (IQR) improved from 0.80 (1–0.6) logMAR preoperatively to 0.2 (0.6–0.1) logMAR at the last available postoperative follow-up visit (*p*<0.001). Post-surgery, BCVA improved in 31 out of 36 eyes (86.1%), while it worsened in 1 eye (2.8%) and remained unchanged in 4 eyes (11.1%), respectively. The mean (±SD) CHW, minimum MH diameter, and base diameters were 534.5 (±207.9) μm, 363.7 (±162.6) μm, and 961.0 (±354.1) μm, respectively. The CHW consistently exceeded the minimum MH diameter and was smaller than the base MH diameter (Fig. 2). Also, all three variables had a moderate to substantial linear relationship (Fig. 2).

**Table 1:** Clinical and imaging characteristics of patients undergoing idiopathic macular hole surgery

Age, years, mean (±SD)	68.3 (±11.7)
Gender, male/female	15/21
Eye, right/left	17/19
Preoperative BCVA, logMAR, median (IQR)	0.80 (0.6–1)
Axial length, mm, mean (±SD)	23.8 (±1.0)
Lens status, no eyes (%)	
Phakic	27 (75%)
Pseudophakic	19 (25%)
MH stage*	
II	2 (5.6%)
III	3 (8.3%)
IV	31 (86.1%)
*OCT parameters*	
MH minimum diameter, μm, mean (±SD)	363.7 (±162.6)
MH base diameter, μm, mean (±SD)	961.0 (±354.1)
Choroidal Hypertransmission width, μm, mean (±SD)	534.5 (±207.9)
EZ absence, no eyes (%)	14 (38.9%)
ELM absence, no eyes (%)	10 (27.8%)

*Based on the IVTS subclassification for MH

*BCVA* best-corrected visual acuity, *ELM* external limiting membrane, *EZ* ellipsoid zone, *IQR* interquartile range, *IVTS* international vitreomacular traction study group, *MH* macular hole, *SD* standard deviation

Table 2 illustrates the results of univariable analysis for factors associated with postoperative BCVA, adjusting for follow-up time. Preoperative MH minimum diameter (for a 10-μm increase, estimate (SE): 0.009 (0.003) logMAR, *p*=0.003), MH base diameter (for a 10-μm increase, estimate (SE): 0.003 (0.001) logMAR, *p*=0.032), CHW (for a 10-μm increase, estimate (SE): 0.008 (0.002) logMAR, *p*<0.001), and delta CHW (for a 10-μm increase, estimate (SE): 0.013 (0.005) logMAR, *p*=0.009) were the only factors significantly associated with postoperative BCVA. CHW explained the highest proportion of variance (*R*^2^ 0.35), followed by minimum MH diameter (*R*^2^ 0.24), delta CHW (*R*^2^ 0.19), and MH base diameter (*R*^2^ 0.14). Upon variable standardization, CHW emerged as the factor with the greatest impact on postoperative BCVA (β (SE), 0.568 (0.137)), followed by minimum MH diameter (β (SE), 0.471 (0.147)), delta CHW (β (SE), 0.421 (0.152)), and base diameter (β (SE), 0.360 (0.161)).

**Table 2:** Univariable linear regression models for factors associated with postoperative BCVA and BCVA change

Variable	Postoperative BCVA		BCVA change	
	*Estimate (SE)*	*p-value*	*Estimate (SE)*	*p-value*
Age, per decade	0.033 (0.044)	0.45	−0.065 (0.089)	0.47
Male gender	−0.118 (0.104)	0.26	−0.123 (0.213)	0.57
Baseline lens status (ref: pseudophakia)	−0.025 (0.117)	0.83	−0.225 (0.234)	0.34
Preoperative BCVA, logMAR	0.155 (0.078)	0.054	N/A	N/A
Follow-up time, months	−0.039 (0.025)	0.14	−0.095 (0.052)	0.08
MH stage IV (ref: II and III)	−0.006 (0.147)	0.97	−0.448 (0.287)	0.13
Preoperative ELM absence	−0.063 (0.114)	0.59	0.071 (0.232)	0.76
Preoperative EZ absence	−0.121 (0.106)	0.26	0.213 (0.216)	0.33
Preoperative cysts	−0.019 (0.223)	0.93	0.206 (0.450)	0.65
Preoperative MH minimum diameter, per 10 μm	0.009 (0.003)	**0.003**	−0.006 (0.006)	0.34
Preoperative MH base diameter, per 10 μm	0.003 (0.001)	**0.032**	−0.006 (0.003)	0.06
Preoperative CHW, per 10 μm	0.008 (0.002)	**<0.001**	−0.003 (0.005)	0.57
Preoperative delta CHW, per 10 μm	0.013 (0.005)	**0.009**	0.004 (0.011)	0.71

Estimates for continuous variables are intended for 1-unit increase unless specified otherwise. All models were adjusted for follow-up time to account for uneven follow-up

*BCVA* best-corrected visual acuity, *CHW* choroidal hypertransmission width, *ELM* external limiting membrane, *EZ* ellipsoid zone, *MH* macular hole, *SE* standard error

**Fig. 3:**
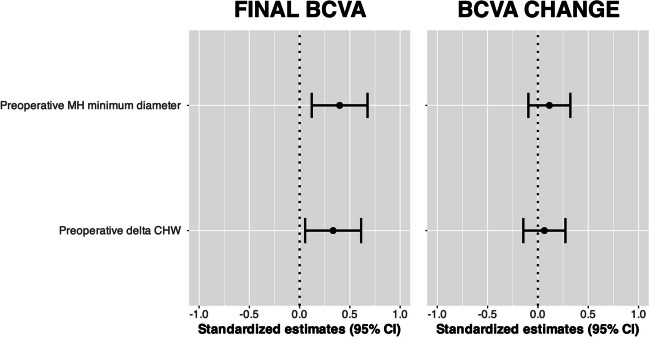
Forest plot for factors associated with final BCVA (left panel) and BCVA change (right panel). Dots and bars indicate standardized point estimates and 95% confidence intervals, respectively. BCVA, best-corrected visual acuity; CI, confidence interval; CHW, choroidal hypertransmission width; MH, macular hole

Figure 3 presents the results from the standardized multivariable model, which included both the MH minimum diameter and delta CHW. Preoperative minimum diameter (β (SE), 0.398 (0.142); *p* = 0.008) and delta CHW (β (SE), 0.333 (0.142); *p* = 0.025) were significantly associated with final postoperative BCVA. Similar results were obtained with the use of normalized delta CHW in the multivariable model (Supplementary Figure 3). The results remained consistent even when preoperative BCVA and the preoperative absence of EZ were incorporated into the standardized multivariable model (Supplementary Table 1). None of the study variables was significantly associated with BCVA change in either the univariable (Table 2) or the multivariable models (Fig. 3, Supplementary Figure 3).

## Discussion

This study investigated preoperative clinical and OCT-derived factors associated with the visual outcome after successful MH closure. We found that MH minimum diameter and delta CHW were associated with the postoperative visual prognosis.

Anticipating surgical outcomes in patients with MH is a significant aspect of retinal surgery. Preoperative MH size is an established predictive factor for postoperative visual results; our findings are in agreement with a multitude of studies showing that smaller MHs have a better prognosis [3, 4, 21]. There is no consensus on the best way to measure MH size, and various OCT metrics have been proposed to evaluate the MH linear dimension, area, volume, and configuration [22–25]. In this study, we included two metrics to inform the MH size, specifically minimum and base diameter. These metrics, which are straightforward to calculate and can be measured on single-line scans, are widely used in clinical practice and classification schemes [6]. We found that the MH minimum diameter was a stronger predictor of postoperative functional outcome than the base diameter, in agreement with previous studies [2]. In a retrospective cohort study, Roth and colleagues [2] evaluated multiple OCT-morphologic parameters and found that minimum linear diameter had the best correlations with MH closure and postoperative VA.

In this study, we investigated whether choroidal CHW has a prognostic role in patients undergoing MH surgery. Although choroidal hypertransmission has been described in a multitude of retinal conditions, such as AMD [16, 17], MFC [18], punctate inner choroidopathy (PIC) [26], multiple evanescent white dot syndrome (MEWDS), and myopic traction maculopathy with outer lamellar MH [19, 27], this OCT biomarker has less well been characterized in idiopathic MH. Recently, Mehta and colleagues [20] observed a positive correlation between CHW and visual acuity. However, the study did not answer the question of whether CHW provided any extra value compared to minimum MH diameter in prognosticating visual acuity recovery.

We found that CHW was associated with postoperative visual outcomes after successful MH surgery and affected postoperative visual recovery more than all other study variables, including MH minimum diameter. We believe that CHW in the setting of MH may have distinctive components. Within the MH aperture, light penetration may be increased because of the absence and lateral displacement of the retinal tissue overlying the RPE. CHW and minimum diameter were highly correlated, with the former increasing as the latter increased. Hence, some of the ability of CHW to explain postoperative VA results may be related to the MH size itself. However, we found that choroidal CHW was consistently larger than MH minimum diameter and extended beyond MH margins. We termed this the mismatch between CHW and MH diameter as delta CHW, and we hypothesize this may be a sign of photoreceptor dysfunction and damage, especially at the level of the EZ-ELM complex, as suggested by a recent study [8]. Neuroretinal detachment from the RPE layer leads to profound modifications to the RPE-photoreceptor interface [8, 28]. Photoreceptors undergo progressive structural damage, with debris shedding into the subretinal space and shortening, distortion, and vacuolation of outer segments up to the point where outer segments appear as empty sacks at the tip of cilium [29]. RPE cells lose their apical villi and the apical-basal polarization, protrude with their cytoplast in the subretinal space, and proliferate the RPE monolayer with multi-layered areas or protrusion into the subretinal space [28]. Macrophages, monocytes, and granulocytes migrate into the subretinal space, usually acellular [28]. Retinal reattachment following MH surgery involves the regeneration of cones’ outer segments, production of the interphotoreceptor matrix, restoration of RPE polarization and villi, and restarted functional relationships among all these structures [28]. The restoration of macular anatomy following MH surgery may occur over many months [9, 30], and patients undergoing successful MH surgery may require up to 2 years to reach their full visual potential [31].

To test the hypothesis photoreceptor damage as measured by delta CHW may be a prognostic biomarker for postoperative visual recovery, we also ran models with delta CHW. We found that delta CHW was associated with postoperative visual outcomes. Still, when delta CHW and MH minimum diameter were included in the same multivariable model, delta CHW impact was less prominent than MH minimum diameter. These findings suggest that both MH size and photoreceptor dysfunction, as measured by delta CHW, may inform the prognosis for patients undergoing MH surgery, with the former having greater importance. We believe CHW may represent a more informative prognostic index than MH size because it includes information from both MH size and outer retina dysfunction. Furthermore, CHW quantification is straightforward and could be automated in future algorithms, [32] potentially accessible on commercially available OCT machines soon.

Other variables previously linked to visual acuity recovery were not confirmed in our study. Disruption of outer retinal hyperreflective bands, including EZ and ELM, have been related to visual outcomes in patients undergoing MH surgery [33] or suffering from many other retinal diseases, including diabetic macular edema [34], age-related macular degeneration [35], and retinal vein occlusion [36]. In our study, preoperative EZ and ELM status were not significantly associated with postoperative BCVA values. Previous studies [3, 4, 21] have found that better preoperative visual acuity was associated with better postoperative visual results. However, our study did not corroborate this finding.

Other study limitations should be acknowledged. This study has a short follow-up time, with the majority of eyes having less than 1 year of observation. Previous research [31] indicates that visual acuity can continue to improve for up to 2 years after MH surgery. Therefore, the follow-up duration in our study might not be sufficient to fully observe the complete recovery of visual acuity postoperatively. The study’s small sample size may have resulted in underpowered analyses. Readers are encouraged to evaluate the point estimates alongside their 95% CI to understand if a certain piece of analysis may be underpowered. A nonsignificant covariate with wide 95% CIs spanning widely around an estimate of zero may indicate that a particular analysis is not powered enough to detect a true association; on the other hand, a significant covariate with wide 95% CIs may indicate a true association but with uncertainty in its exact magnitude. The multivariable models showed limited ability to predict postoperative BCVA, explaining less than one-third of the total variance. This suggests that other, unaccounted-for variables might be influencing a significant portion of the visual recovery that remains unexplained. We did not include some variables of potential interest, such as symptom duration or other OCT-based parameters. Previous studies [3, 4, 21] have shown that MH duration prior to surgery is associated with the postoperative functional outcome, with more recent MHs having better visual recovery. Other, more complex macular hole indices, reflecting MH area, volume, and configuration, have been described. Still, they are not commonly used in clinical practice, may be difficult to calculate, and did not unequivocally prove to be any better than more straightforward and clinician-friendly indices, such as minimum and base MH diameter [2, 37]. CHW is a bidimensional entity, but we only measured its linear diameter at the point where the macular hole dimension is the greatest; further research is needed to evaluate whether CHW area, linear diameter at other locations (e.g., MH margins), or hyperreflectivity intensity has better prognostic value. As we only included patients with successful idiopathic MH surgery, the prognostic role of CHW may not be generalizable to eyes with MHs secondary to other conditions or where surgery was not successful. Similarly, since most of the eyes included in this study were classified as stage IV, the findings may not be broadly applicable to cases of less advanced MHs. We also excluded patients operated with the inverted flap technique, and results may not be generalizable to this technique.

In conclusion, this study provides evidence that OCT CHW is an independent prognostic factor associated with postoperative visual acuity in patients undergoing successful surgery for idiopathic MH surgery. Within a single index, CHW may summarize information regarding the MH size and degree of photoreceptors and RPE dysfunction. Further research is needed to validate this finding across diverse patient populations.

### Supplementary information

ESM 1 (TIFF 32739 kb)

ESM 2 (TIFF 26158 kb)

ESM 3 (TIFF 18878 kb)

ESM 4 (DOCX 59 kb)
