# Supplementary figures and images for: Choroidal hypertransmission width on optical coherence tomography: a prognostic biomarker in idiopathic macular hole surgery
Source: Graefes Arch Clin Exp Ophthalmol. 2024 Mar 26;262(8):2481–9. doi: 10.1007/s00417-024-06427-8 (PMC11271440; doi:10.1007/s00417-024-06427-8)

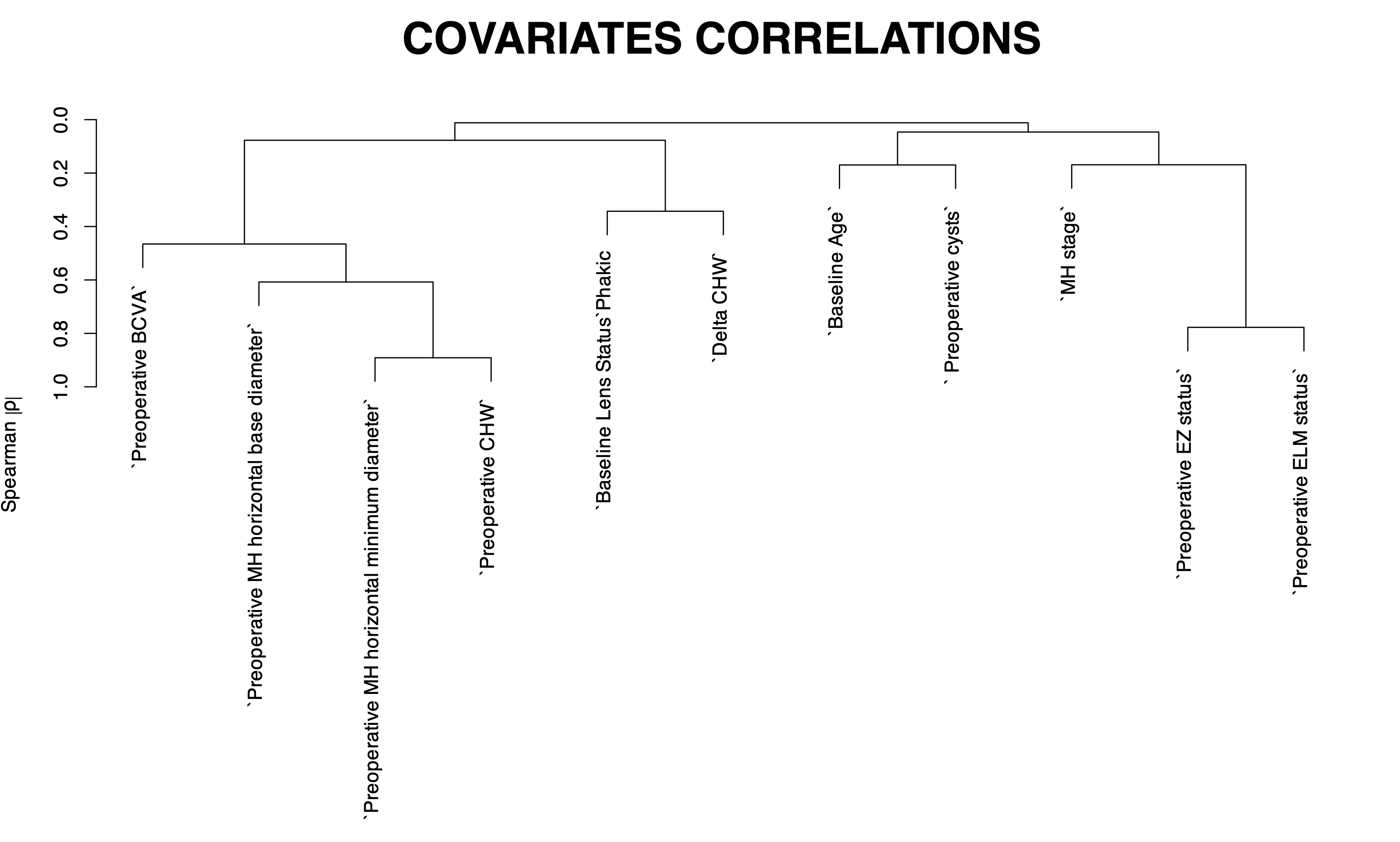

Supplement: Supplementary file 1 — (TIFF 32739 kb) [file 417_2024_6427_MOESM1_ESM.tiff]

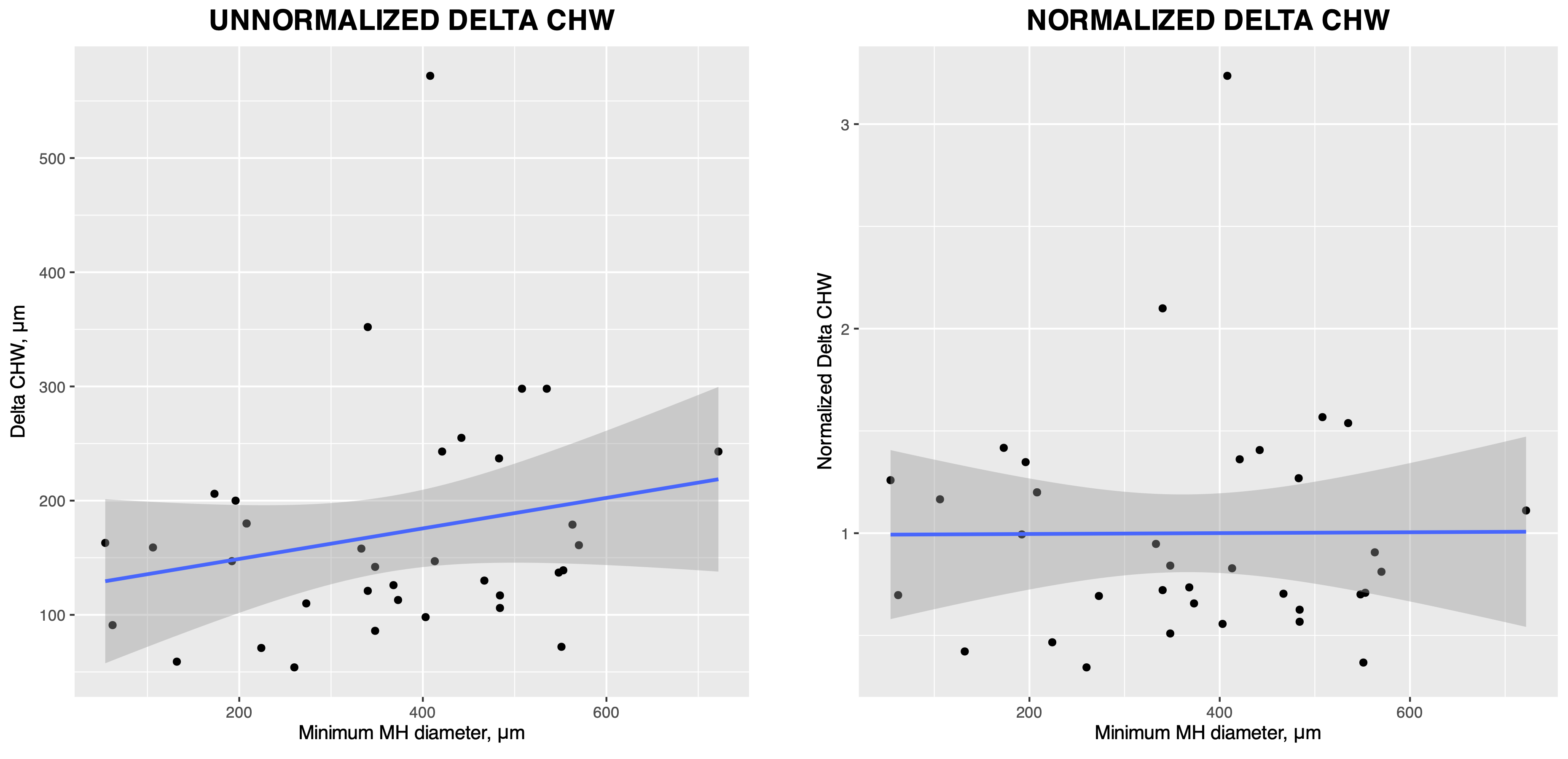

Supplement: Supplementary file 2 — (TIFF 26158 kb) [file 417_2024_6427_MOESM2_ESM.tiff]

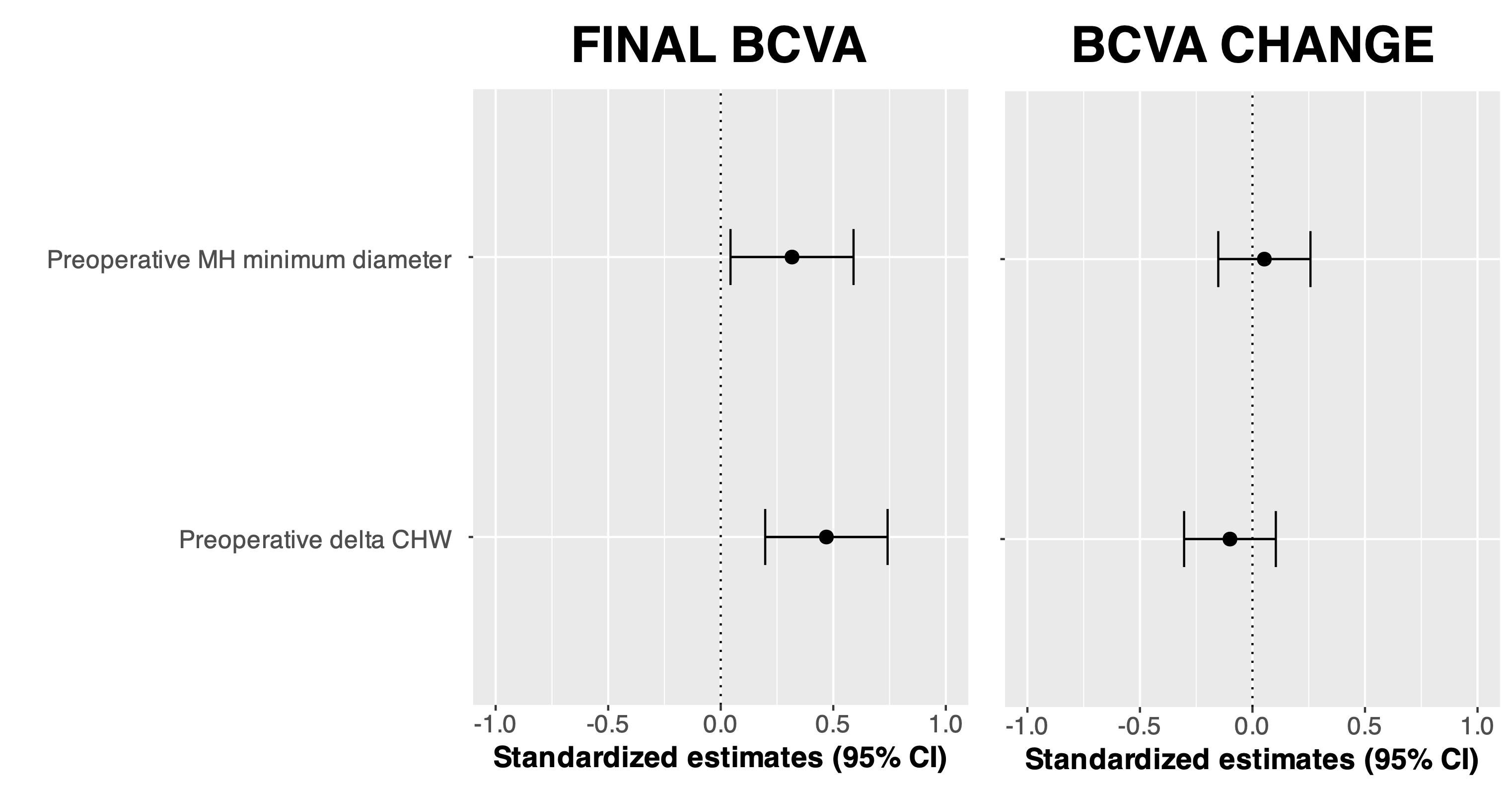

Supplement: Supplementary file 3 — (TIFF 18878 kb) [file 417_2024_6427_MOESM3_ESM.tiff]

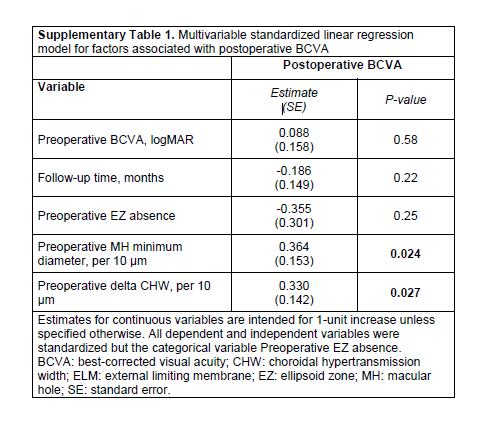

Supplement: Supplementary file 4 — (DOCX 59 kb) [file 417_2024_6427_MOESM4_ESM.docx]
